# Syncytin-1, syncytin-2 and suppressyn in human health and disease

**DOI:** 10.1007/s00109-023-02385-6

**Published:** 2023-10-19

**Authors:** Petra Priščáková, Michal Svoboda, Zuzana Feketová, Juraj Hutník, Vanda Repiská, Helena Gbelcová, Lajos Gergely

**Affiliations:** Institute of Medical Biology, Genetics and Clinical Genetics, Faculty of Medicine, Comenius University Bratislava, Sasinkova 4, Bratislava, 811 08 Slovak Republic

**Keywords:** Syncytin-1, Syncytin-2, Suppressyn, HERVs, Preeclampsia, Gestational trophoblastic disease

## Abstract

In this review, we summarized the results of experimental and clinical studies about three human endogenous retroviruses and their products—syncytin-1, syncytin-2, and suppressyn in human physiology and pathophysiology. We summed up the described connection with various pathological processes and diseases, mainly with pregnancy-induced hypertensive diseases such as preeclampsia, oncogenesis, gestational trophoblastic disease, and multiple sclerosis. Supposed mechanisms of action and the potential of clinical applications are also described.

## Introduction

**Fig. 1:**
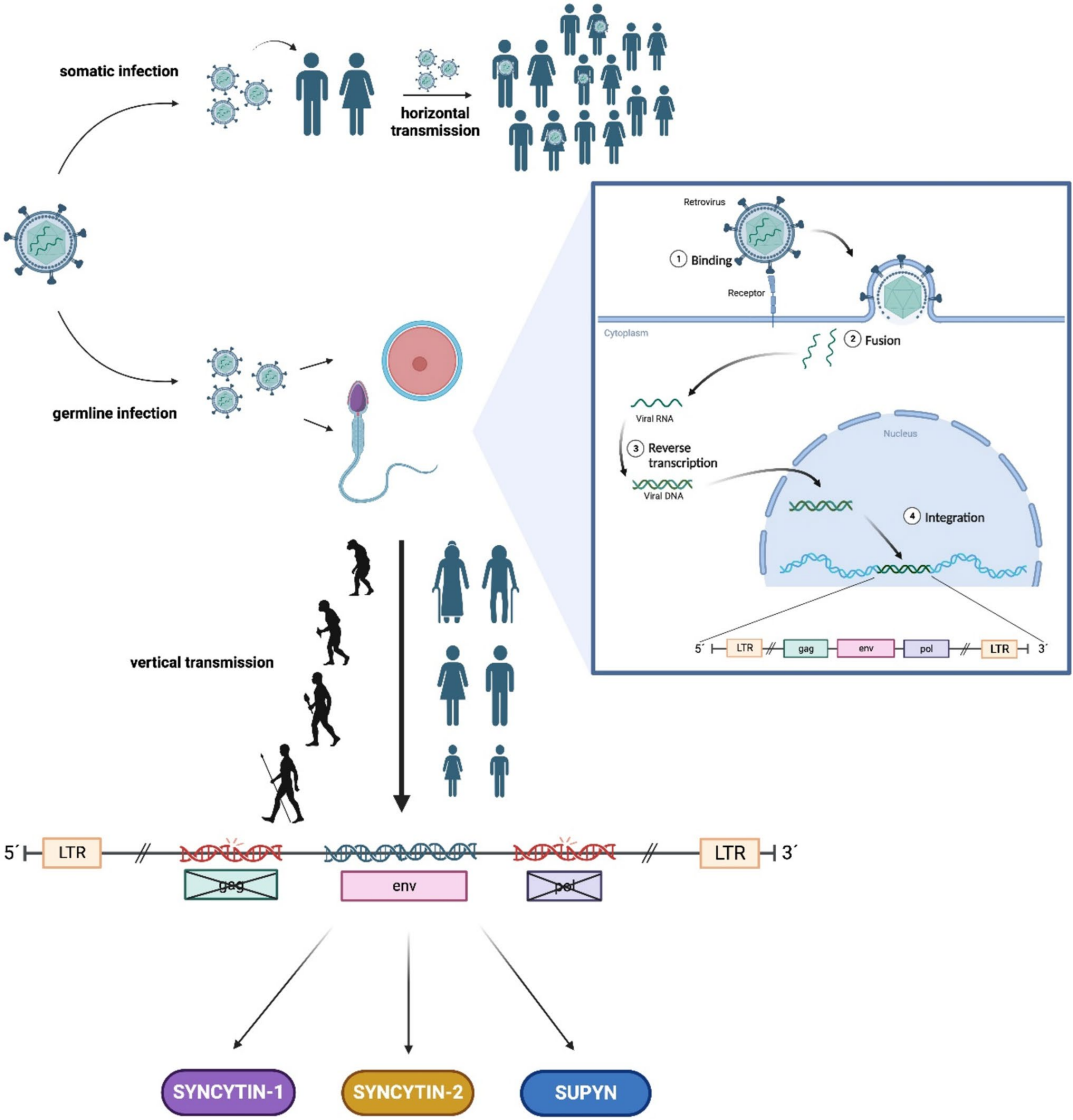
Evolutionary origin of human endogenous retroviral genes (HERVs). Retroviruses can infect somatic cells of the host and the virus can then spread in the population (horizontal transmission). Retroviral infection of host germ cells potentially results in the integration of viral DNA into the host genome. Integrated sequences display typical proviral structure with two LTR flanking both ends and *gag*, *env*, *pol* viral genes. In the case of germ cell infections, these sequences can be vertically inherited in Mendelian fashion (vertical transmission) and preserved in some form during evolution to provide a selective advantage. Mutations in proviral sequences have caused these viruses to become unfunctional. However, in rare cases during evolution, the ORFs of some of the viral genes have become conserved and adopted several physiological functions, mainly in placenta formation. LTR, long terminal repeat; SUPYN, suppressyn. Created in biorender.com

Infection of a host cell by an exogenous retrovirus potentially results in an integration of retroviral DNA into the host cell’s genome. In case of germ cell infection, the inserted retroviral DNA can be afterward vertically inherited in a Mendelian fashion (Fig. 1), and retroviral DNAs are then titled as endogenous retroviruses (ERV) [1]. The genomes of all vertebrates include the aforementioned viral sequences. Actually, 8% of the human genome consists of HERVs (human endogenous retroviruses) [2]. Being fragments of ancient retroviral infections, HERVs display a typical proviral structure consisting of two long terminal repeats (LTRs) flanking the proviral internal portion, composed of the viral genes *gag*, *pol*, and *env*. LTRs contain elements used for the initiation and termination of transcription such as enhancers, promoters, and polyadenylation signals [3]. The *gag* gene codes group-specific antigens and structural proteins such as the nucleocapsid, matrix, and capsid proteins. The *pol* gene codes for the viral replication enzymes including reverse transcriptase, protease, ribonuclease, and integrase essential for the transcription of the viral RNA into double-stranded DNA and integration of the DNA produced by reverse transcriptase into the host’s genome. Lastly, the *env* gene codes for viral envelope glycoprotein are important for receptor recognition and membrane fusion [1, 4]. In most cases, the open reading frames (ORFs) in HERVs are interrupted by a wide variety of mutations like deletions, frameshift, or nonsense mutations. In rare situations, intact ORFs from endogenized ERVs have been conserved for millions of years of evolution. Preservation of functional gene across multiple generations suggests that such gene provides a strong selective advantage for a species during evolution. Env proteins that are involved in trophoblastic differentiation are named syncytins. Syncytins in humans are syncytin-1 and -2, coded by env genes captured 25 and 40 million years ago, respectively. Interestingly, retroviral *env* genes have been repeatedly independently captured (from different retroviral lineages) during evolution to supposedly fulfill critical physiological functions in the placenta of several mammalian species [5, 6] (syncytin-a and syncytin-b in *Muroidea*, syncytin-Ory1 in *Leporidae*, syncytin-Car1 in some of the carnivores, syncytin-Rum1 in *Ruminantia*, syncytin-Opo1 in opossum and related species) [5, 7]. Several more *env* genes or parts of HERVs, that share some but not all of the characteristic features of syncytins, are expressed in the human placenta (for example *ERV3-1*, *ERVV-1*, *ERVPb1*, *HERV-E*) [8].

While a subset of these HERV env share individual features with syncytins, including placental expression, fusogenic activity, ISD presence, and preservation by natural selection, none meet all of these criteria. This suggests that they could be the remnants of ancestrally co-opted syncytins. They are progressively losing their function in some primates, maybe as a consequence of the incorporation of new retroviral elements, such as *ERVFRD-1* and *ERVW-1*, which functionally replaced them [8]. However, ORFs of these *env* remain mainly intact which suggests their roles in other physiological processes.

Our review mainly focuses on the role of three retroviral *env* products, syncytin-1, syncytin-2, and suppressyn, as the most studied HERVs in human health and diseases. They play a crucial role in the formation of the placenta. Domestication of these particular HERVs represents a fascinating example of convergent evolution via the co-option of a retroviral gene for a key biological function in reproductive biology [9] (Fig. 1). On the other hand, their ectopic and aberrant expression can lead to the development of several disorders.

## Physiological functions of syncytins in human

Syncytins, syncytin-1, and syncytin-2 in humans are coded by *env* from retroviral elements incorporated in the genome that possess crucial roles in the differentiation of trophoblast during the formation of the placenta. They regulate the creation of syncytiotrophoblast, hence the name syncytins.

Syncytin-1 is a 73 kDa glycosylated protein composed of 538 amino acids. It is encoded by the *ERVW-1* gene, localized in locus 7q21.2. This gene was acquired by primates around 25 million years ago [10]. Syncytin-1 consists of two main subunits, the surface (SU) and the transmembrane (TM) subunits. SU contains the signal peptide, while TM contains the fusion peptide, the immunosuppressive domain (which is part of a larger ectodomain), and the transmembrane domain [11, 12] (Fig. 1)*.*

Syncytin-2 is a human *envelope* protein with fusogenic activity, encoded by a gene from the HERV-FRD family—also known as *ERVFRD-1*. *ERVFRD-1* has been functionally conserved in primates for more than 40 million years which suggests the strong effect of purifying selection [13]. Amino acid sequence analysis of fusogenic FRD *env* with the typical hydrophobic profile revealed the consistency with the typical characteristics of retroviral envelopes: FRD *env* is represented by the canonical cleavage site with the consensus R/K-X-R/K-R amino acid sequence, which is located between SU and TM protein subunits. TM subunit contains a hydrophobic domain representing the fusion peptide, a transmembrane domain, and a putative immunosuppressive domain. SU subunit includes the canonical “CWLC” domain, which plays a role in SU-TM interactions in *env* proteins [5, 13]. The subunits’ functions are analogous to syncytin-1’s, as well as the mechanism of cell fusion.

**Fig. 2:**
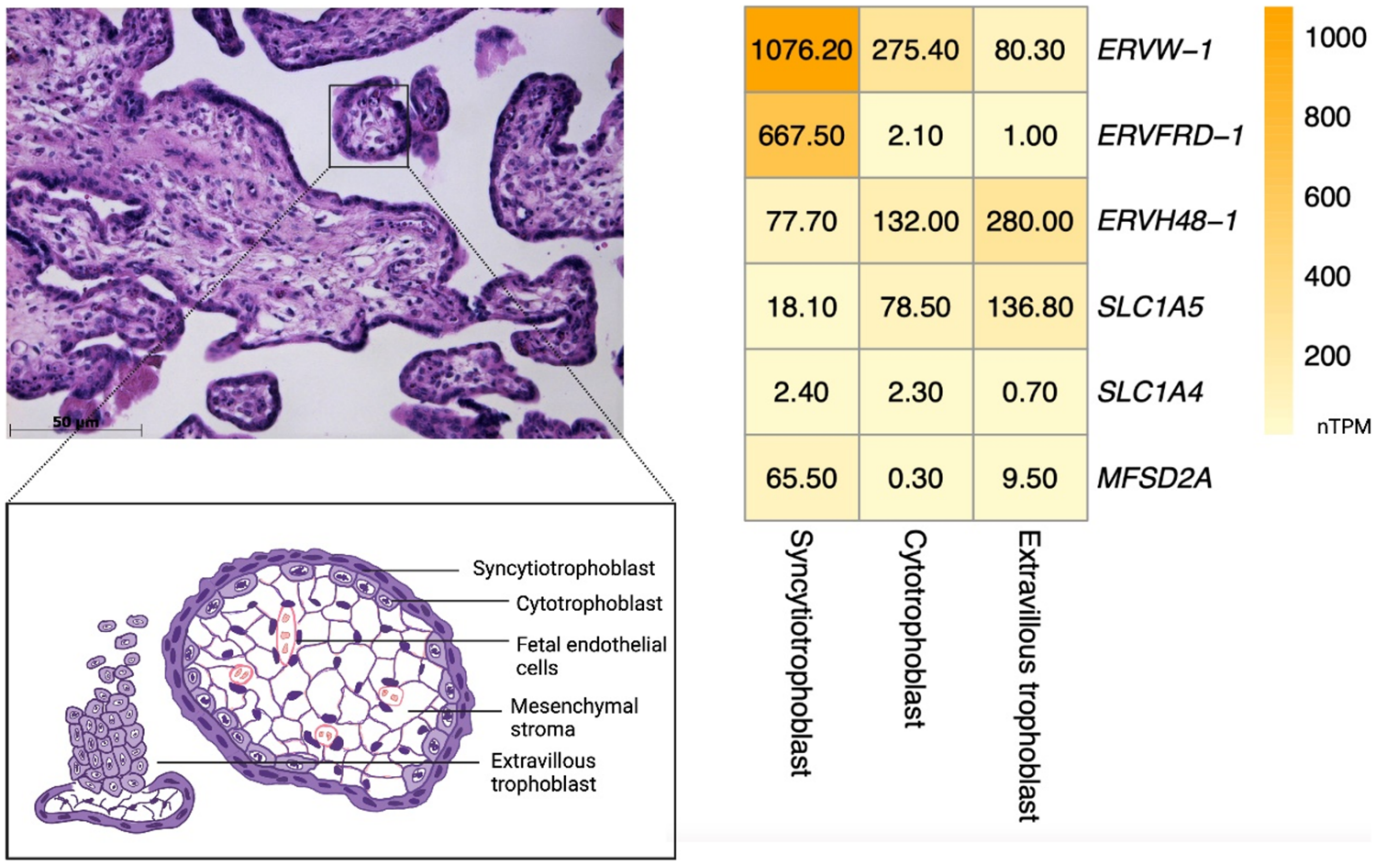
Expression levels of selected HERVs and their receptors in placental tissue. Chorionic villi and trophoblast populations in a 28-gestational-week placenta with a schematic cartoon representation of chorionic villus (left). Expression levels of genes coding syncytin-1 (*ERVW-1*), syncytin-2 (*ERVFRD-1*), suppressyn (*ERVH48-1*), and their receptors (*SLC1A5*, *SLC1A4*, *MFSD2A*) in trophoblast populations (right); nTPM, normalized protein-coding transcripts per million. Expression data from proteinatlas.org. The placenta was stained by hematoxylin and eosin. An image of the placenta was taken by light microscope Axio Vert. A1 in software Axio Vision 4.8 (Zeiss) by Lajos Gergely, MD (Institute of Medical Biology, Genetics and Clinical Genetics, Faculty of Medicine, Comenius University Bratislava, Bratislava, Slovak Republic). Created in biorender.com

The role of syncytins in human physiology involves essential fusogenic and nonfusogenic processes like the formation of multinucleated syncytium (Fig. 2), cell cycle regulation, and possibly apoptosis [14–16].

**Placenta formation:** Syncytins are expressed almost exclusively in the placenta [13, 17, 18] and have fusogenic activity [19–21]. Therefore, syncytium formation is induced solely by syncytin-1 and syncytin-2. Syncytins’ genes are predominantly expressed in the trophoblasts, where protein fusogenic activity has been co-opted for the development of the placental syncytiotrophoblast. It originally functioned in favor of the virus and provided receptor-mediated virus—cell fusion alternatively evolving toward cell—cell fusion. Infection of target cells by retroviruses is initiated by the fusion of the retroviral envelope with the cytoplasmatic membrane of target cells. The fusion is possible thanks to the interaction of env with a target receptor. Most infection of cells by retroviruses leads to the expression of the retroviral env protein on the surface of infected cells and to syncytialization of the infected cells with cells expressing the viral receptor [22]. Syncytins are such env proteins and their SU subunit interacts with the target receptor, which leads to a conformation change of the TM subunit. This conformation change exposes the fusion peptide. The fusion peptide located on the N-terminus consists of hydrophobic amino acids which enable initial penetration of the target cell membrane. The fusion active core structure brings cytoplasmatic membranes of interacting cells into close proximity resulting in membrane fusion [23].

In 2000, syncytin-1 was identified for the first time in the syncytiotrophoblast layer of human placental villi [19] and also its receptor—SLC1A5/ASCT2/RDR (a neutral amino acid transporter and type D mammalian retrovirus receptor) [21]. When the syncytin-1 gene was transfected into COS cells (fibroblast-like cell lines derived from monkey kidney tissue), syncytia formed consisting of many aggregated nuclei surrounded by an extended cytoplasm [1, 19]. The same discovery suggested that syncytin-1 might be the key factor in trophoblast fusion.

The SU domain of syncytin-1 binds to the placental vascular endothelium and affects placental vascularization. Knock-down of syncytin-1 in BeWo and HTR-8/SVneo cells deactivated the PI3K/Akt/mTOR signaling pathway, which eventually led to decreased expression of *VEGF* and *PLGF* which altogether led to placental hypoxia [24].

Several studies have detected *ERVFRD-1* transcripts in the placenta [13, 25, 26] and primary human trophoblast culture [27]. At the cellular level, the localization of syncytin-2 was observed in the cytoplasmic membrane of primary trophoblast cells [27]. More specifically, using in situ hybridization, the syncytin-2 localization was restricted to villous cytotrophoblast [25] and this observation was consistent with the results of Malassiné et al. obtained by the immunostaining [17] (Fig. 2). Syncytin-2 protein interacts through its specific receptor MFSD-2 (major facilitator superfamily domain containing 2) [28]. In cultured primary human trophoblast cells, a gradual increase in the relative level of syncytin-2 mRNA was observed with prolonged cultivation time (cultivation was performed up to 96 h). On the contrary, the level of syncytin-1 mRNA was rapidly elevated after 24 h of cultivation and decreased after that. Syncytin-2 signals were mostly detected at the membrane in place of cell-to-cell contact, probably where syncytin-2 interacts with its specific receptor to induce fusion [27]. Based on the knock-down of syncytin genes and the impact on cell fusion, Vargas et al. suggested that syncytin-2 may play a more important role in cell fusion events than syncytin-1 does [27]. It is an interesting observation, as the level of syncytin-1 transcript is higher in trophoblast cells compared to syncytin-2 transcript level based on the data from proteinatlas.org [29]. It can be deduced that the effect of syncytin-1 is restricted not only in amount-dependent manner, but by another regulatory mechanism, like the existence of competitive inhibitor suppressyn. Syncytin-2 has a strong impact on placental development; however, the process of trophoblast cell fusion requires the cooperation of several *env* proteins [27].

During pregnancy, the transcription level of *ERVFRD-1* in the first trimester placentas was about 10-fold lower compared to *ERVW-1* [26]. Also, a progressive decrease of *ERVFRD-1* transcription level was observed as pregnancy proceeded, which was in contrast with the *ERVW-1* expression pattern. Kudaka et al. assume that this decreasing pattern with gestational age might be a consequence of the altered ratio of cytotrophoblasts to syncytiotrophoblasts and/or other placental cells [25, 26]. Based on the above-mentioned data, we could propose that syncytin-2 has a crucial role in the formation of syncytiotrophoblast in the very early (perhaps pre-villous) stages of placenta development.

**Feto-maternal immune tolerance:** Tolerance of the fetus by the immune system of the mother is a key factor for the maintenance and successful outcomes of the pregnancy. The syncytiotrophoblast continuously interacts and monitors surrounding dendritic cells, macrophages, T-lymphocytes, and decidual natural killer cells (dNK). This constant monitoring regulates the immunosuppressive state that is absolutely required to prevent rejection [30]. The exact molecular mechanisms involved in syncytin-1-mediated feto-maternal immune tolerance are not elucidated [31]; it is assumed that the presence of surface factor (likely different from the receptor needed for syncytin-mediated fusion) is essential. The highly conserved part of the syncytins’ immunosuppressive domain (ISD) is probably able to activate extracellular signal-regulated kinase (ERK1/2), mitogen-activated protein (MAP) kinases, and other cellular effectors [32]. However, more mechanistic studies are needed to better understand how this envelope region can modulate the immune response and which immune cell populations are targeted by the ISD of the syncytins [32]. Additionally, it was proposed that ISD, except for immunosuppressive function, also contributes to the formation and stabilization of the disulfide bond [31]. The disulfide bond covalently links the SU and TM subunits of syncytins, which is crucial for syncytin stability and plays a role in the conversion of the receptor-bound complex to a fusion-active form. For gammaretroviruses, deltaretroviruses, and type-D betaretroviruses, the bond forms between a CXXC motif in SU and a highly conserved CXnCC motif in TM. ISD is adjacent to the CX6CC motifs in all gamma-type Envs, where syncytins also belong [31].

Syncytin-1 is also present in placental exosomes that enable syncytin-1 to reach and interact with target cells of the maternal immune system. Further, syncytin-1 successfully inhibits the production of cytokines TNF-α, IFN-γ, and chemokine CXCL10 in human leukocytes [12]. Based on the mentioned observations, syncytin-1 is possibly an important player in the process of reaching the feto-maternal immune tolerance and is actively secreted into the maternal circulation in the form of placenta-derived exosomes.

During normal pregnancy, the second trimester is characterized by inhibition of the Th1 and activation of the Th2 immune response. Syncytin-2 ISD activates MAP kinase and leads to inhibition of Th1 cytokines (TNF-α, IFN-γ, and IL-2), which extends the observation of the impact of syncytin-1 ISD on the inhibition of Th1 cytokine secretion [32]. Both syncytin-2 and syncytin-1 were detected on the surface of placenta-derived exosomes. Exosomes provide a mechanism by which syncytin-2 (and also syncytin-1) can modulate the immune response at a distance and in different regions surrounding the placenta [33, 34].

Stable and functional syncytiotrophoblast probably contributes to the regulation of placental expansion. The syncytiotrophoblast sheds aggregates of nuclei into the maternal circulation during pregnancy which can trigger the maternal immune tolerance of the fetus. In the case of an abnormal rate of shedding, like when syncytiotrophoblast is not stable, the mother may suffer from excessive invasion and chimerism [35].

**Regulation of the cell cycle:** The syncytiotrophoblast is primarily post-mitotic under physiological conditions. Therefore, mononucleated cells must exit the cell cycle before undergoing cell fusion. It was observed that only G0-arrested cells were able to fuse, but restriction of *ERVFRD-1* expression is needed. Overexpression of *ERVFRD-1* resulted in the formation of syncytia which was unstable and had functional defects [35]. Expression of *ERVFRD-1* is regulated by p21 and GCM1. Together they bind to the promoter of the syncytin-2 gene and induce its transcription. This is the direct connection of cell-cycle machinery and fusogens, as it seems that restriction of *ERVFRD-1* expression in G0-arrested cells is crucial for the formation and maintenance of healthy syncytiotrophoblast [35]. Syncytin-1 regulates the G1/S transition. Overexpression of *ERVW-1* in CHO cells promoted cell proliferation and knockdown blocked G1/S transition [36]. Syncytin-1 can maintain optimal cytotrophoblastic “pool” by inducing cell proliferation and then fusion of the cytotroblastic cells [36].

**Gamete fusion:** Syncytin-1 is present in human sperm cells, dominantly localized in the sperm head and around the equatorial segment. The receptor ASCT-2 is present in the acrosomal region and the sperm tail. *ASCT-2* is also expressed in oocytes [37]. Based on these results, we could hypothesize that syncytin-1 plays an important role in human fertilization by facilitating the fusion of gametes.

**Resistance to retroviral infection:** Syncytin-1 is recognized with ASCT2 receptor [38]. Gammaretroviruses feline endogenous retrovirus RD-114, baboon endogenous retrovirus, *Reticuloendotheliosis virus A* (REV-A), and spleen necrosis virus (SNV) and betaretroviruses simian retrovirus serotypes 1–5 also use ASCT2 to enter the host cells [38, 39]. Cellular resistance to retroviral infection is possible by receptor interference, which means that env of different retroviruses are recognized by the same receptor. Primary infection of a cell with a virus prevents secondary infection of the cell by another virus from the same interference group by blocking the receptor or even by inducing downregulation of receptor expression [39, 40]. Expression of *ERVW-1* and production of syncytin-1 induce cellular resistance to SNV in a cell line normally sensitive to it which can explain the inability of human cells to be infected by SNV and REV-A [40, 41].

**Osteoclast generation:** The generation of osteoclasts through the fusion of mono-nucleated precursors is a key event of bone physiology and bone resorption is inefficient without osteoclast fusion [42]. The expression of *ERVW-1* and its receptor *ASCT2* was proved in differentiating osteoclasts in vitro. These in vitro findings were confirmed by immunohistochemical staining in human iliac crest biopsies. Further, syncytin-1 inhibition also inhibits osteoclast fusion [42], which suggests its crucial role in the fusion of osteoclasts.

**Muscle fiber formation:** Myoblast fusion into multinucleated muscle fibers is crucial for human skeletal muscle development. The gene for syncytin-1 and its receptors ASCT-1 and -2 are expressed in human myoblasts and syncytin-1 is involved in myoblast fusion [43], so it was proposed they play a crucial role in the regulation of myoblast fusion. Frese et al. analyzed skeletal muscle biopsies of competitive cyclists. Comparing muscle biopsies from post- with the pre-competitive seasons, they found evidence of increased cell fusion together with the increased expression of gene encoding syncytin-1. Furthermore, they proved that myoblast treatment with anti-syncytin-1 abrogates cell fusion in vitro [44] and demonstrated that syncytin-1 is an essential protein involved in mediating cell fusion in muscles.

## The role of syncytins in various human diseases

**Fig. 3:**
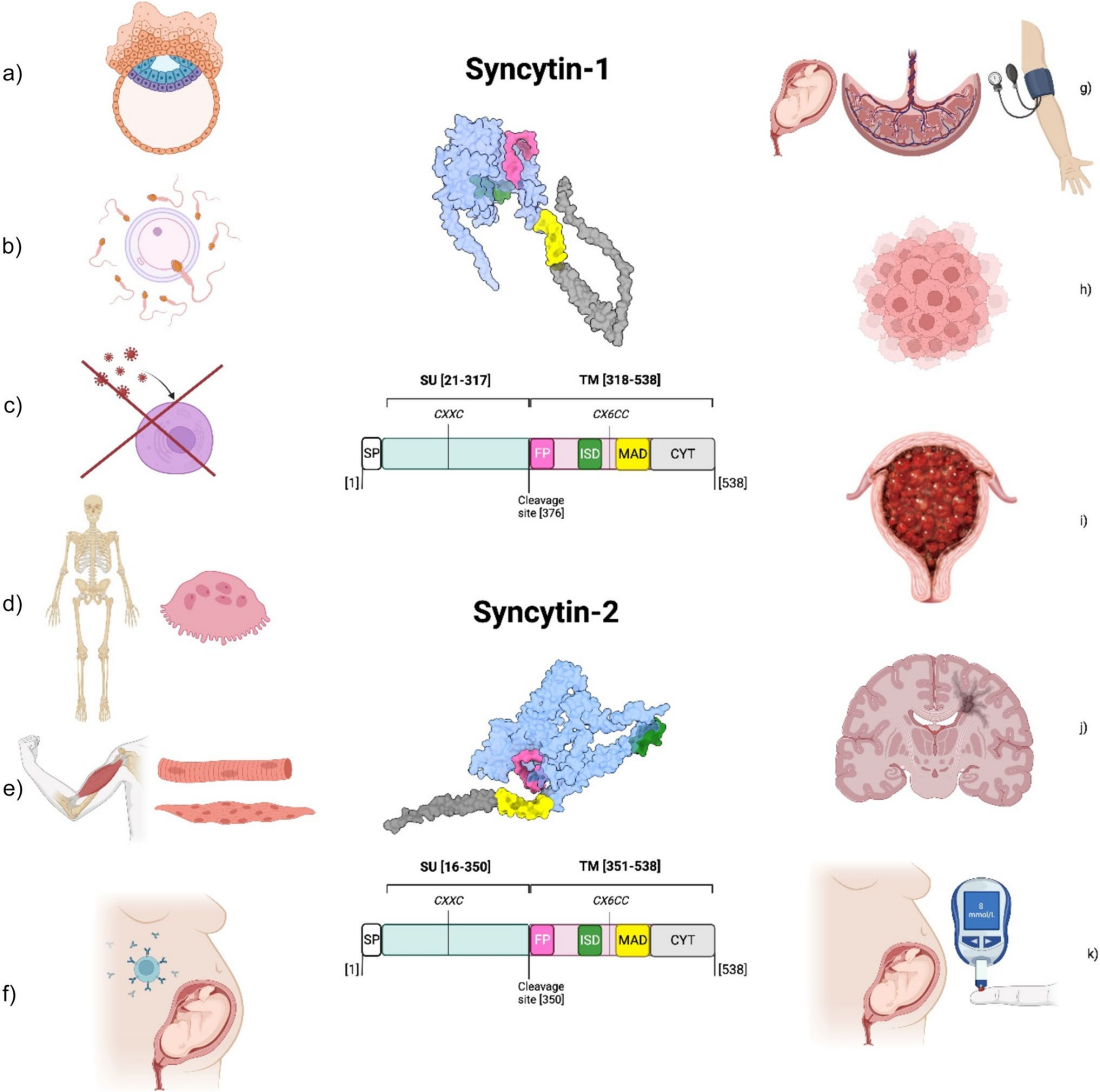
Structure and roles of syncytins in the human body. Syncytin-1 and syncytin-2 are both involved in several physiological (left) and pathological (right) processes. In terms of structure, both genes display SU and TM units together with FP, ISD, MAD, and CYT domains. CXXC and CX6CC motives are essential for the creation of disulfide bonds between TM and SU units in protein. Syncytin-1 is involved in placenta formation (**a**), gamete fusion (**b**), prevention of viral infections (**c**), osteoclast formation (**d**), myotube formation (**e**), and feto-maternal immune tolerance (**f**). Syncytin-2 is involved in placenta formation (**a**) and feto-maternal immune tolerance (**f**). In terms of the pathogenesis of disease, syncytins were found to be involved in (**g**) preeclampsia, (**h**) tumorigenesis, (**i**) gestational trophoblastic disease, (**j**) multiple sclerosis, and (**k**) gestational diabetes. SU, surface unit; TM, transmembrane unit; FP, fusion peptide; ISD, immunosuppressive domain; MAD, membrane anchorage domain; CYT, cytoplasmic domain. Protein structures were generated by AlphaFold [97]. Molecular graphics performed with UCSF ChimeraX (rbvi.ucsf.edu/chimerax). Created in biorender.com

A number of studies have identified alterations of HERV expressions in placental pathological contexts. HERVs and syncytins are associated not only with preeclampsia (PE) and gestational trophoblastic disease but also with malignancies and multiple sclerosis (MS) (Fig. 3).

**Gestational complications:** Preeclampsia (PE) is a serious multifactorial disease with onset during pregnancy. PE is the second most common cause of mortality in pregnant women. It is characterized by elevated blood pressure and proteinuria after 20 weeks of gestation [45]. Defective formation of the placenta plays an important role in the pathogenesis of this disease. Invasion and replacement of endothelial cells in the spiral arteries of the inner third of the myometrium by intermediate trophoblast are frequently altered in PE. Moreover, abnormal syncytiotrophoblast differentiation is also observed in preeclamptic placentas, with an increased proportion of syncytial knots and an early hypermaturation of chorionic villi. In fact, syncytins are responsible for the physiological progress of these processes [46]. There is a considerable amount of literature regarding reduced syncytins’ gene expression and the pathogenesis of PE. Vargas et al. described a deficient cellular fusion of trophoblast cells in preeclamptic placentas when compared to controls [47]. Lee et al. reported dramatically reduced *ERVW-1* expression in placentas with PE and also abnormal localization of proteins toward the apical syncytiotrophoblast microvillous membrane as opposed to its normal location on the basal syncytiotrophoblast cytoplasmic membrane [48]. Many other observations of reduced syncytin-1 mRNA and protein levels in preeclamptic placentas were reported [49–52]. Interestingly, *ERVFRD-1* expression was more significantly altered than *ERVW-1* [47]. Additionally, the results suggested a correlation between the level of *ERVFRD* expression and the severity of PE disease [47]. Syncytin-2 quantity, detected in serum-derived exosomes, is also reduced in women with PE compared to healthy pregnant women [33].

Hua et al. investigated whether some specific SNPs (single nucleotide polymorphisms) may potentially alter the expression or syncytins’ structure and therefore be associated with an elevated risk of PE [53]. From the group of selected and analyzed tag SNPs in *ERVW-1* and *ERVFRD-1* genes, only the rs9393931 variant of *ERVFRD-1* showed a significant association with PE development. In comparison with controls, there was a significantly higher T allele frequency of this mentioned SNP in PE cases. Surprisingly, homozygotic state (TT) was extremely significantly associated with PE. To sum up, the authors declared that the TT genotype has an increased risk of PE development, unlike the other genotypes [53]. Variant rs9393931 is localized in the 3′-UTR region, so it is not affecting the protein sequence. However, variants in this region can affect the processing of mRNA, mRNA stability, and translation efficiency. However, no significant correlation of the variant with the clinical severity of PE was detected, besides a slight difference in blood pressure between high-risk and low-risk genotypes (not significant) [53].

The exact role of syncytins in the pathogenesis of PE is not known yet. We could hypothesize that the lower expression of syncytin-1 in preeclamptic placentas is not necessarily the cause of the pathologic process, but it can be a consequence of it. It is known that local and systematic inflammation is considered a central factor for PE development. Inflammation takes part in vascularization, hypoxia, and dysregulation of immune cell response. Hypoxia leads to a lower level of syncytin-1 [15]. Syncytin-1 contributes to placental development in multiple ways including syncytial renewal through fusion of cytotrophoblast and modulation of maternal immune system response to fetal cells [16]. A lower amount of syncytin-1 could lead to the disruption of these processes, resulting in the abnormal placentation typical of preeclampsia, which could exacerbate the already existing inflammation and hypoxia. Exposure to air pollution can increase the risk of adverse pregnancy outcomes such as hypertensive disorders in pregnancy (like preeclampsia) or fetal growth restriction [54]. The study found that exposure to respirable particulate matter with a diameter of less than 10 µM was associated with an increased concentration of plasmatic extracellular vesicles (EV) containing syncytin-1 (Sync-1 + EV). Increased Sync-1 + EV levels were associated with lower level sVCAM-1 (a marker of endothelial dysfunction). It can be hypothesized that increased release of this EV might counterbalance the proinflammatory effect of particulate matter exposure [54].

Detyrosination of microtubules is necessary for the promotion of trophoblast syncytialization. Regulation of tubulin detyrosination was compromised in preeclamptic trophoblast syncytialization, which led to the accumulation of syncytin-2 in the cytoplasm of cells. Enhancement of detyrosination led to enrichment of syncytin-2 on the cell membrane and increased syncytium formation. Dysregulation of tubulin tyrosination potentially leads to the onset of preeclampsia [55].

Anyway, syncytin-1 could be a hopeful predictive marker of PE. Several research groups detected that conditions associated with placental pathology such as preeclampsia lead to differences in total EV quantities in maternal plasma and that the fraction of syncytiotrophoblast-specific EVs (containing syncytin-1) is significantly changed [56–58]. Levine et al. found an increased quantity of EV in the maternal plasma of preeclamptic females; however, a significantly lower fraction of EV contained syncytin-1 compared to the plasma of pregnant women without preeclampsia [58]. Preeclampsia and preeclampsia-like syndrome of pregnant women with COVID have similar clinical and laboratory characteristics, which makes differential diagnostics difficult. Differentiation between these conditions is important because of distinct management [59]. Presumably, the method based on profiling and quantifying EV could distinguish females with preeclampsia, as their EV profiles would be unique and distinct from pregnant females with COVID-19. Development of non-invasive, early predictive tests, is strongly required because they would help to identify patients with the need for preventive steps like the administration of low-dose aspirin [60].

Fetal growth restriction (FGR) is defined as a condition when fetal weight estimated by ultrasound is less than the 10th percentile. FGR is associated with an increased risk of perinatal morbidity and mortality, and the risk of adult diseases is increased at the same time [61]. The regulatory regions of *ERVW-1* and *ERVFRD-1* are hypomethylated in FGR cases compared to cases small for gestational age and appropriate for gestational age. These data suggest a possibility of using the methylation status of syncytin genes as a biomarker in early diagnostics of FGR [61]. Mouse syncytins are not phylogenetically related to human syncytins but they possess similar functions (fusogenic properties and high expression in the placenta) [62]. Knock-out of the syncytin-a gene in mice led to embryonic lethality and FGR possibly due to the disruption of nutrient exchange at the maternal–fetal interface and decreased glucose transporter levels, which emphasized pleiotropic functions of syncytins in placenta development and in sustaining viable gestation [63]. Recurrent spontaneous abortion (RSA) is defined as two or more consecutive spontaneous abortions before 28 weeks of gestation [64]. The etiology and pathogenesis of RSA are often not elucidated. Hypermethylation of *ERVW-1* and lower levels of syncytin-1 were detected in RSA villous tissue. Syncytin-1 has an anti-apoptotic function [14]; hence, its decreased level can trigger apoptosis and the occurrence of RSA, similar to the knock-out of syncytin-a in mice. It is proposed that the assessment of the methylation status of *ERVW-1* can be a predictive and diagnostic marker of RSA [64]. *ERVFRD-1* expression was detected in proliferative cytotrophoblast cells in the placenta in some cases of RSA. This finding suggests that fusion of trophoblast cells in the wrong stage of the cell cycle may lead to pregnancy abnormalities. It is not clear if unstable trophoblast or abnormal endocrine function, both caused by overproduction of syncytin-2, contribute to RSA [65].

There is less known about syncytins in gestational diabetic placentas which are characterized by villous immaturity and increased fibrinoid necrosis in the placenta [66]. Soygur et al. noted that the expression levels of *ERVFRD-1* and also the gene for MFSD-2 receptor are reduced in gestational diabetic human term placentas [66]. Further functional studies are needed to answer whether altered gene expression for syncytin-2 and MFSD2 might either be the consequence or cause of gestational diabetes.

**COVID-19:** In the last years, syncytin-1 has received high public attention mainly due to misinformation connecting COVID-19 mRNA vaccination and infertility. Authors of the above-mentioned reasoned their claim with the presumed production of anti-syncytin-1 antibodies due to vaccination. The assumption was based on that a similar sequence of five amino acids can be found in corona spike protein (length of protein 1273 amino acids) and syncytin-1 (length of protein 538 amino acids) which represents only 0.75% homology [67]. The overall amino acid identity and similarity between syncytin-1 and the SARS-CoV-2 spike protein are only 8.8% and 15.8%, respectively [68]. For instance, the antibody developed against MSRV for the treatment of multiple sclerosis has an overall homology of 81% with syncytin-1 and still, it is not affecting syncytin’s functionality regarding its function in the placenta [69]. Additionally, no evidence of anti-syncytin-1 antibody was found following COVID-19 mRNA vaccination [70], there is no evidence of cross-reactivity of anti-SARS-CoV-2 spike protein antibodies with syncytin-1 [68], and several studies confirmed no association between an increased rate of infertility in females or males and COVID-19 vaccination [71, 72].

However, Balestrieri et al. found an interesting association between COVID-19 and Env protein from the *HERV-W* family. They described high expression of *HERV-W env* in the leukocytes of COVID-19 patients but not in those of healthy controls. The percentage of lymphocytes expressing *HERV-W* envelope correlated with inflammatory markers and pneumonia severity in COVID-19 patients, reflecting the respiratory outcomes during hospitalization [73]. These findings suggest that SARS-CoV-2 can induce aberrant and ectopic expression of *ERVW-1*, which can contribute to the immunopathology of COVID-19 such as dysregulation of innate immunity associated with hyper-inflammation [73].

**HIV:** According to the study of Tang et al., syncytin-1 has an important role in the creation of HIV reservoirs in the placenta—concretely in the trophoblast—of infected pregnant women. HIV uses env proteins for interaction with CD4 receptors and co-receptors on target cells to enter them and initiate infection [74]. However, HIV has been detected in cells that are not expressing CD4 on their surfaces, such as trophoblast. Syncytin-1 and HIV-1 envelope glycoprotein (GP160 env) share similar structural profiling, both are proteolytically cleaved into two structural units, surface and transmembrane unit. Their core structures (N- and C-terminal heptad repeats) essential for membrane fusion have high sequence similarities, 44% and 62%, respectively [23]. It was found that HIV is able to hijack other viral envs for entry into CD4-negative cells. They proved that syncytin-1 triggers the fusion of trophoblast with HIV-infected T-cells, which is independent of HIV Env and CD4. This fusion results in HIV transmission into CD4-negative cells and the creation of an HIV reservoir in the placenta, hampering a radical cure for HIV infection [74].

**Cancer:** Oncogenesis is a complex multistep process hypothesized to be the result of cooperation between genetic and environmental factors, including viral infections. The oncogenic properties of exogenous retroviruses are well known. In fact, retroviruses were originally identified as causative agents of transmissible tumors in chicken and mice. Exogenous retroviruses are able to induce carcinogenesis directly via viral oncogene—*v-onc* or indirectly by insertional mutagenesis and disruption of tumor suppressor genes [75]. The effect of HERVs, and particularly syncytins, is on the other hand less certain. HERV envelope proteins have been suggested to support tumorigenesis through the exact same biological activities that made them persist in the human genome—fusogenicity and modulation of the immune response [76, 77]. The immune system is one of the most essential defenses of the host against tumors. HERV envelope proteins may promote carcinogenesis through immunosuppression in a similar way as they help in fetomaternal immune tolerance—through the inhibition of cytokine production [12]. Cytokines may inhibit tumor development; therefore, their reduction in circulation might facilitate cancer progression [78, 79]. Fusion of cells is a physiological process that can be found in certain tissues like muscles or the placenta, but is frequently observed in pathological conditions, such as cancer or inflammation. Multiple studies reported elevated levels of the *HERV-W* group RNA and proteins in human malignancies. Bjerregaard et al. were among the first to describe over synthesis of syncytin-1 in breast cancer and also provided definite evidence that syncytin is involved in mediating fusion between breast cancer cells and endothelial cells suggesting spontaneous, syncytin-mediated fusion in breast cancer [80]. Liu et al. performed syncytin-1 immunohistochemistry on 130 samples of endometrial carcinoma [81]. They used Kaplan–Meier analysis to assess the overall survival according to syncytin-1 expression. The mean survival time of patients was shorter if a high *ERVW-1* expression level was detected in the tumor tissue. The 5-year cumulative survival rate was 32.8% in patients with endometrial carcinoma and high *ERVW-1* expression and 60.7% in those with low expression of *ERVW-1* [81]. The authors also analyzed the effects of syncytin-1 overproduction (reached by plasmid transfection) in vitro. They found that the overexpression of *ERVW-1* can promote cell proliferation, cell cycle progression, and the migration and invasion of endometrial carcinoma tumor cells. On the other hand, suppression of *ERVW-1* expression inhibited cell proliferation and apoptosis in vitro [81]. The expression of *ERVW-1* substantially elevated the expression levels of epithelial-mesenchymal transition-related genes (*VIM*, *CDH1*, *SLUG*, *ZEB1*), but significantly decreased the expression of epithelial markers (N-cadherin, snail) [81]. Promotion of epithelial-mesenchymal transition—and in this way—the increase of tumor cells’ invasiveness offers a potential explanation of the worse prognosis associated with high *ERVW-1* expression. The *ERVW-1* gene is physiologically expressed in some types of tissues above mentioned. However, in the majority of tissues, *ERVW-1* expression is epigenetically controlled by methylation of CpG islands in the promoter region [82]. This phenomenon is particularly important as aberrant activity would surely lead to complications. This case was demonstrated by Benesova et al. in seminomas and was attributed to the hypomethylation of the *ERVW-1* promoter region resulting in aberrant expression and possible cancer-promoting effect in testicular germ cell tumors [82]. Hypomethylation of *ERVW-1* has also been detected in non-small cell lung cancer and a high level of syncytin-1 has been associated with a worse prognosis [83]. Syncytin-1 promoted hepatocellular carcinoma progression and doxorubicin resistance via the inflammation-activated MEK/ERK pathway [84].

All of these studies highlighted that syncytin-1 not only mediates trophoblast cell fusion in the placenta but may also play a role in the fusion of host-cancer cells and cancer-cancer cells and the regulation of the cell cycle. The role of syncytin-1 in oncogenesis is still not certain; however, its effect on cell proliferation, migration, and invasion as well as cell cycle arrest are very attractive and promising for future research and validation of the syncytin-1 changes as a prognostic or diagnostic biomarker.

Gestational trophoblastic disease is a group of benign, premalign, and malign pregnancy-associated pathologies characterized by abnormal proliferation of trophoblast [85]. Bolze et al. performed immunohistochemical localization of syncytin-1 in hydatidiform moles, gestational trophoblastic neoplasia, and control placentas [86]. They also assessed the transcription levels of *ERVW-1* by quantitative PCR. According to the results of immunohistochemistry, the amount of syncytin-1 was enhanced in the apical part of the syncytiotrophoblast of hydatidiform moles compared to normal placentas. Complete hydatidiform moles with further malignant transformation had a higher staining intensity of syncytin-1 surface unit, but the transcription level of *ERVW-1* was not different from complete hydatidiform moles without malignant transformation. *ERVW-1* transcription was down-regulated in hydatidiform moles and gestational trophoblastic neoplasia compared to control placentas [86]. The variations of syncytin-1 protein localization likely reflect altered functions of syncytin-1 in the premalignant context of complete hydatidiform moles. The reduced transcription of *ERVW-1* in gestational trophoblastic diseases suggests a dysregulation mechanism in a malignant context [85, 86]. Huang et al. demonstrated in vitro rapid cell fusion in human gestational choriocarcinoma cell lines via up-production of syncytin-1 by inducing *ERVW-1* expression with forskolin [36]. An interesting part of the experiment was siRNA induced knockdown of *ERVW-1*, which resulted in impaired cell growth as well as a blocked G1/S transition phase of the cell cycle. Increased *ERVW-1* expression triggers placental trophoblast cell cycle changes through the downregulation of p15 and upregulation of CDK4, which promotes the G1/S transition [36].

During pregnancy, syncytin-1 can probably repress the maternal immune system. On the other hand, studies indicate that an elevated amount of syncytin-1 in the brain is involved in the development of neuropsychological diseases by triggering abnormal inflammation [87]. Several activating pathways are proposed to explain the involvement of syncytin-1 in neuropsychiatric diseases, namely, elevation of a nitric oxide level in the glial cells, activation of the TLR4/CD4 pathway, activation of the TLR3 signal pathway, or induction of cytotoxic T lymphocytes. These inflammatory abnormalities could lead to neuronal damage and apoptosis of neuron cells, which play crucial roles in neuropsychiatric diseases such as schizophrenia and MS [87].

**Multiple sclerosis:** Multiple sclerosis (MS) is one of the most common non-traumatic neurological diseases in young adults [88]. It is a chronic progressive disease, accompanied by demyelination, inflammation, axonal damage, and progressive neurologic dysfunction [89]. There is a growing body of research on the role of HERVs in the etiopathogenesis of MS. The first remarks of possible retroviral particles of endogenous origin in MS patients date back to the early 1990s [90]. The cDNA sequences derived from particle-associated RNA were then assigned to a “multiple sclerosis-associated retrovirus” (MSRV) belonging to the HERV family [90, 91]. An interesting fact is that MSRV and syncytin-1 are closely related as they are both members of the HERV-W family and the only structural difference between them is a 12-nucleotide insertion in the transmembrane moiety of MSRV [92, 93]. Retroviral env proteins are believed to be able to cause neuroinflammation, neurodegeneration, and endoplasmic reticulum stress. A number of studies present the important role of syncytin-1 in the development of MS. *ERVW-*1-encoding RNA levels are elevated in CNS of MS patients. Antony et al. reported higher levels of syncytin-1 in glial cells of patients with acute demyelination MS [94]. In the same study, they managed to insert syncytin-1 into a virus that was able to infect astrocytes. This form of virus was then injected into the brains of mice. Overexpression of syncytin-1 in astrocytes boosted the release of redox reactants that were cytotoxic to oligodendrocytes. Two weeks after injection, the mice developed symptoms of MS [94, 95]. Syncytin-1 is particularly overexpressed in the glia of MS patients, and its possible pathogenic mechanism is attributed to the production of cytokines and reactive oxygen species followed by oligodendrocyte injury and endoplasmatic reticulum stress, that leads to damage of nearby cells. Anthony et al. have shown that syncytin mediates the production of proinflammatory molecules such as iNOS, IL-1β, and redox reactants in vitro, that at higher levels cause oligodendrocytes death, demyelination, and overall damage to the brain [94]. Higher levels of syncytin-1 in the brain of MS patients, compared to controls as well as multiple evidence showing that syncytin-1 induces inflammation, oxidative stress, and reduced myelin production in the brain, are proving probable relevance of syncytin-1 in the pathophysiology of MS. Recently, a phase 2, double-blinded, 48-week trial was carried out in relapsing–remitting MS. The trial assessed the efficacy and safety of temelimab, a *HERV-W* envelope-neutralizing monoclonal antibody. Although temelimab failed to show an effect on features of acute inflammation but demonstrated preliminary radiological signs of possible anti-neurodegenerative effects [96].

The role of syncytin-1 is more thoroughly studied both in physiological and pathological context in humans. Based on the known properties of syncytin-2, we can hypothesize that syncytin-2 has also profound roles in human health, but more research in this area needs to be done to confirm this.

## Suppressyn

Another placental protein, belonging to the group of HERVs, is called suppressyn (SUPYN). It is suggested, that SUPYN negatively regulates cell fusion; therefore, it functionally differs from the other two HERV proteins [39]. SUPYN coding sequence (*ERVH48-1*) originates from the *env*-region of the HERV-member family—*HERV-Fb1* [98] and it is transcribed both in the early and late placenta [25, 26, 39]. The sequence *HERV-Fb1* appears at several positions in the genome; however, there is only one locus, 21q22.3, where the full-length coding sequence is present but in reverse orientation. SUPYN protein consists of 160 amino acids and its DNA and amino acid sequence is highly conserved over simian evolution, which suggests that it is not a pseudogene. The predicted 18 kDa translation product is part of SU (surface subunit) and does not comprise presumed N-glycosylation sites in contrast with *ERVW-1 (8)*; nevertheless, a single O-linked site is included [99]. Furthermore, the transcription product contains a premature STOP codon (truncating product before SU-TM cleavage site) and the translation product includes a putative signal sequence. It is typical for *HERV env* genes to contain immunosuppressive domain (ISD); however, SUPYN does not include either the TM or ISD [39, 100].

### Physiological functions of suppressyn

SUPYN was identified in placenta and trophoblast (TB) cell line cultures [39]. The protein was detected both in villous and extravillous trophoblasts (EVT). The positive staining signal was observed in progenitor cytotrophoblast cells (CTB) and intermediate trophoblast (ITB) (in the contact regions of anchoring villi and maternal decidua) (Fig. 2). Robust expression was observed mainly at sites, where the endothelial cells are replaced by ITB in maternal blood vessels [99]. This observation indicates that SUPYN could be involved in the maternal spiral arteries remodeling process. In general, the preferential expression was detected in unfused cells, suggesting its antifusogenic potential. However, the weak signal was also notable in syncytiotrophoblast (ST), which could potentially be the remaining protein in the previously unfused cytoplasm of CTB cells [99]. In terms of pregnancy progress, the relative transcription level of *ERVH48-1* was almost constant during pregnancy (in the second trimester, the expression was slightly but insignificantly higher) [26]. Sugimoto et al. distinguished two types of SUPYN. The cell associated (intracellular) isolated from cell lysates, and other, the secreted form of SUPYN, isolated from the supernatant [39]. siRNA knock-down of the *Fb1* gene promotes cell fusion in BeWo cells as well as the differentiation process [39]. Moreover, using the immunoprecipitation method, they found that SUPYN binds to the syncytin-1 receptor (ASCT-2), which indicates the potential mechanism of SUPYN function via binding the ASCT-2 receptor [39]. Both intracellular and secreted forms of SUPYN bind to ASCT-2, thereby inhibiting cell fusion in TB cell lines and primary placental tissues [39]. The cell-associated SUPYN showed full inhibition of cell fusion, whereas the secreted form exhibited incomplete but detectable inhibition (~ 30%) [39].

Detected forms of SUPYN intracellular and secreted indicate that it could play a role in intracellular, paracrine, and autocrine pathways. Sugimoto et al. performed an experiment, in which HTR8 cells (first-trimester human trophoblast cells) were transiently transfected with a vector to overexpress SUPYN. Overexpression of SUPYN resulted in the development of the smaller molecular weight smear of endogenous ASCT-2 that occurred in western blots, which indicates protein degradation, ubiquitination, or some alterations in the maturation of endogenous ASCT-2 [99]. It was found that SUPYN alternated the N-linked glycans on ASCT2. It can be suggested that intracellular SUPYN can induce alternations in ASCT2 glycosylation likely during intracellular processing of SLC1A5 for cell surface expression [98] which is a possible SUPYN-mediated way of the cell fusion rate regulation.

SUPYN can play paracrine role in receptor interference. Cell culture assays showed that SUPYN could restrict infection by mammalian type D retroviruses which also supports a hypothesis that retroviral elements are endogenized to provide host immunity and genome defense [101]. We can hypothesize that released SUPYN can inhibit viral infection of cells in which it is not expressed. That could be the possible reason for the fairly robust ability of the human placenta to delimit vertical transmission of a subset of exogenous pathogens [39]. Since SUPYN is missing ISD, it is not able to regulate the response of the maternal immune system, even if is released into the bloodstream. SUPYN possibly regulates cell fusion of trophoblast cells in which it is expressed or in a nearby area in an autocrine manner by blocking the receptor for syncytin-1. SUPYN derived from villous cytotrophoblast could also inhibit the fusion of syncytiotrophoblast debris with maternal local and peripheral *ASCT2*-expressing somatic cells [99], such as maternal spiral arteries, arterioles, veins, venules, and glands during their invasion and remodeling of these structures in early pregnancy [98]. SUPYN presence can explain how it is possible, that regardless of high *ERVW-1* expression in the placenta, is this process still highly regulated.

In addition, based on transcriptional and translational analysis, it was suggested that syncytin-1 and SUPYN are both taking part in hypoxia/hyperoxia reactions to syncytia formation in the placenta [39, 99].

### The role of suppressyn in various human diseases

In terms of the pathogenesis of human diseases, there is little known about SUPYN. Kudaka et al. refer to a significantly lower transcriptional level of *ERVH48-1* in placentas with pregnancy induced hypertension (PIH) compared to the normotensive (control) group. As we mentioned before, the same event was observed also in the case of syncytin-1 and syncytin-2 [25]. Sugimoto et al. suggest the potential connection between SUPYN and the etiology of abnormal placentation diseases such as PIH or PE. It can hypothesized that an increased amount of SUPYN leads to abnormal syncytialization and shallow placental invasion typical for preeclampsia [39]. However, these assumptions need to be examined by future investigations [39, 99].

The chromosomal localization of SUPYN overlaps with the Down syndrome critical region, so we could hypothesize that an increased copy number of the SUPYN locus in trisomy 21 affected placentas could be important in the context of abnormal placenta morphology in Down syndrome, which is characterized by delayed maturation of cytotrophoblast cells and reduced syncytialization [102]. Sugimoto et al. proved that the increased copy number of the SUPYN locus in the placenta with trisomy 21 is associated with reduced fusion of cytotrophoblast cells into syncytiotrophoblast, with increased SUPYN transcription, translation, and secretion [102]. Further, they reported an increased concentration of secreted SUPYN in the serum of women with pregnancies affected by Down syndrome, which could be potentially applied in prenatal screening [102].

## Conclusion

**Fig. 4:**
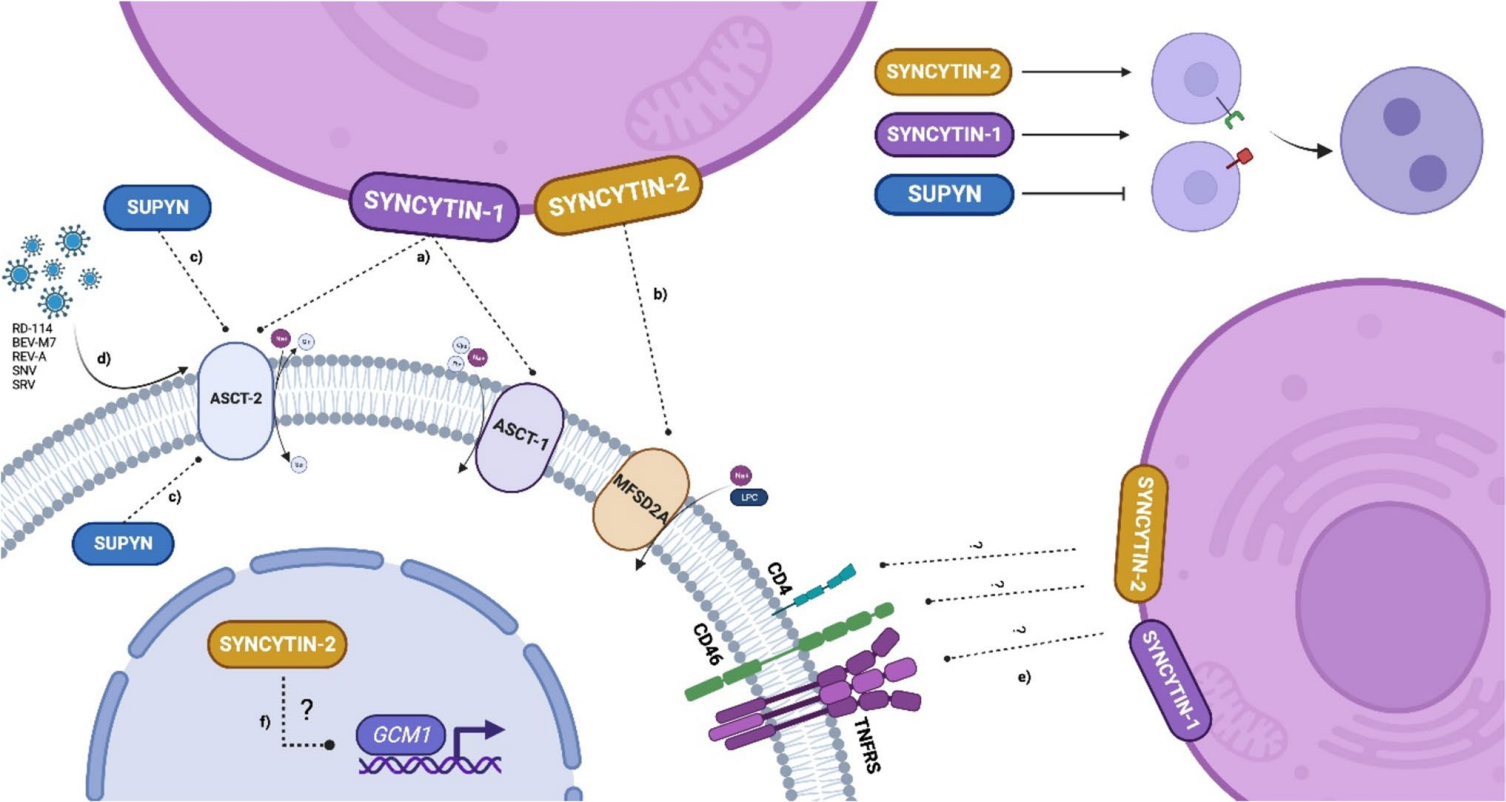
Confirmed and potential interactions of syncytin-1, syncytin-2, and suppressyn. Syncytin-1, located in the cellular membrane, interacts with both ASCT-1 and ASCT-2 receptors, resulting in cell–cell fusion. **a** Syncytin-2, also localized in the cellular membrane, binds to the MFSD2A receptor, promoting cell–cell fusion. **b** Suppressyn, found in both intracellular and secreted forms, binds to the ASCT-2 receptor, inhibiting cell–cell fusion. **c** ASCT receptors function mainly as sodium-coupled amino acid transporters, whereas MFSD2A acts as a sodium-dependent LPC symporter. ASCT-2 also represents a binding site for gammaretroviruses feline endogenous retrovirus RD-114, baboon endogenous retrovirus, Reticuloendotheliosis virus A (REV-A), spleen necrosis virus (SNV), and betaretroviruses simian retrovirus serotypes 1–5 (SRV). **d** Syncytins potentially interact with CD4, CD46, and TNFRS receptors, which can result in activation of various signaling cascades. **e** Inside the nucleus, syncytin-2 potentially interacts with the GCM1 transcription factor. **f** These interactions were predicted by the https://string-db.org/ algorithm. Functions of ASCT-1, ASCT-2, and MFSD2A receptors were derived from https://uniprot.org/. Created in biorender.com

The presence of *ERVW-1*, *ERVFRD-1*, and *ERVH48-1* (coding syncytin-1, syncytin-2, and SUPYN proteins, respectively) in the human genome is the result of ancient retrovirus infections followed by integration into the nuclear genome and vertical transfer among generations. Selection pressure led to the domestication of these integrated retroviruses which was a crucial moment not only in human evolution. Recently, the *env* gene products of these retroviruses have played important roles in human physiological processes, from which the formation of the placenta and fetomaternal immune tolerance is the most known and investigated. Plenty of molecular associations with other human proteins are proven/predicted (Fig. 4).

Syncytin-1 was the most investigated among these three proteins so it is not surprising that a wide spectra of its physiological functions are known mainly due to its fusogenic and immunologic activities. Syncytin-2 and suppressyn present less investigated proteins so their recently narrow known spectrum of physiological roles needs to be completed. It is clear that for proper development of a placenta, cooperation of syncytin-1 and syncytin-2 is crucial, with the additional help of other HERVs. Suppressyn represents a unique element that can regulate the rate of fusion induced by syncytin-1 and helps to restrict cell fusion in the placenta. It is an interesting question if such repressor exists also for syncytin-2 or if regulation of cell fusion induced by syncytin-2 solely depends on the restriction of syncytin-2 production and function.

Abnormal placentation, pathological gestation, cancer, neurodegeneration, etc., are possibly in some way associated with altered function of these genes. However, whether alterations in the expression of these genes are the cause or the result of pathological processes is not totally clear yet, so potential applications including therapeutical down/upregulation of expression needs further experimental and clinical investigation. A more recent potential clinical application of the knowledge about the association of these genes with various human diseases is the development of biomarkers for differential diagnostic/prognostic/screening purposes. Today, in the era of noninvasive testing using extracellular nucleic acids and secreted microvesicles isolated from the plasma of patients, detection of altered expression of these genes could potentially help in early diagnostics of some human diseases and enable early therapeutic interventions, potentially with a prophylactic character.

The potential of fusogenic activity of syncytins in medical applications is demonstrated by two recent patents. According to these patents, syncytins can be reliably used for targeted delivery of a therapeutic drug to the lungs and skeletal muscle, including a therapeutic gene or a gene encoding a therapeutic drug. Syncytins, in particular, lentiviral vectors pseudotyped with syncytins, can be used to deliver drugs including transgenes in lung alveolar and skeletal muscle cells following systemic administration. This opens new ways for the treatment of pulmonary diseases and myopathies, such as cystic fibrosis and Duchenne muscular dystrophy [103, 104].

## Data Availability

Not applicable.
